# A Piece of the Puzzle—Possible Mechanisms for Why Low Dietary EPA and DHA Cause Hepatic Lipid Accumulation in Atlantic Salmon (*Salmo salar*)

**DOI:** 10.3390/metabo12020159

**Published:** 2022-02-08

**Authors:** Bjørg Kristine Hundal, Esmail Lutfi, Trygve Sigholt, Grethe Rosenlund, Nina Sylvia Liland, Brett Glencross, Nini Hedberg Sissener

**Affiliations:** 1 Department of Feed and Nutrition, Institute of Marine Research, P.O. Box 1870, Nordnes, 5817 Bergen, Norway; Nina.Liland@hi.no (N.S.L.); Nini.Sissener@hi.no (N.H.S.); 2 Department of Nutrition and Feed Technology, Norwegian Institute of Food, Fisheries and Aquaculture Research (Nofima), P.O. Box 210, 1431 Ås, Norway; esmail.lutfi.royo@nofima.no; 3 BioMar AS, Havnegata 9, 7010 Trondheim, Norway; trysi@biomar.com; 4 Skretting Aquaculture Research Centre, P.O. Box 48, 4001 Stavanger, Norway; Grethe.Rosenlund@skretting.com; 5 Institute of Aquaculture, University of Stirling, Stirling FK9 4LA, UK; Bglencross@iffo.com

**Keywords:** lipid metabolism, EPA, DHA, robustness, Atlantic salmon, metabolomics

## Abstract

The present study aimed at elucidating the effects of graded levels of eicosapentaenoic acid (EPA) and docosahexaenoic acid (DHA) on the hepatic metabolic health of Atlantic salmon reared in sea cages. Diets containing 10, 13, 16 and 35 g/kg EPA + DHA (designated diets 1.0, 1.3, 1.6 and 3.5, respectively) were fed in triplicate through a full production cycle from an average starting weight of 275 g to slaughter size (~5 kg). Feeding low dietary EPA + DHA altered the hepatic energy metabolism, evidenced by reductions in tricarboxylic acid cycle intermediates originating from β-oxidation, which was compensated by elevated activity in alternative energy pathways (pentose phosphate pathway, branched chain amino acid catabolism and creatine metabolism). Increases in various acylcarnitines in the liver supported this and indicates issues with lipid metabolism (mitochondrial β-oxidation). Problems using lipids for energy in the lower EPA + DHA groups line up well with observed increases in liver lipids in these fish. It also aligns with the growth data, where fish fed the highest EPA + DHA grew better than the other groups. The study showed that diets 1.0 and 1.3 were insufficient for maintaining good liver metabolic health. However, diet 3.5 was significantly better than diet 1.6, indicating that diet 1.6 might also be suboptimal.

## 1. Introduction

The dietary substitution of fish oil (FO) by vegetable oils (VO) in feed for Atlantic salmon (*Salmo salar)* is a common practice in aquaculture due to the limited availability of FO. This causes a distinct reduction in eicosapentaenoic acid (EPA, 20:5n-3) and docosahexaenoic acid (DHA, 22:6n-3) and typically increases in linoleic acid (18:2n-6), α-linolenic acid (18:3n-3) and oleic acid (18:1n-9). Multiple reports have shown that exchanging FO for some types of VO (such as rapeseed, soy oil and sunflower oil) results in hepatic lipid accumulation in salmon over time [1,2,3,4,5,6,7,8,9,10]. In humans, non-alcoholic fatty liver disease (NAFLD) is strongly associated with obesity, diabetes, dyslipidaemia, cardiovascular disease, hypertension and, ultimately, the metabolic syndrome [11,12]. Demonstrating that hepatic lipid accumulation is detrimental and linked to reduced fish robustness has been difficult, as many trials have been short-term, land-based and/or under controlled conditions. Even so, there are indications that a higher liver lipid content is a symptom of a less robust fish. Salmon kept in large, open sea cages have suddenly experienced large increases in mortality, with the only common denominators between moribund fish being fatty liver combined with increased plasma alanine aminotransferase and alkaline phosphatase levels, both being marker enzymes of liver damage [13]. A trial feeding salmon 2, 10 and 17 g/kg EPA + DHA found significantly higher liver lipid contents in fish fed the two lowest levels of EPA + DHA [2]. Additionally, there were higher mortality rates in fish fed these two diets. Another trial in sea cages found increased mortality combined with a considerably fattier liver (10 vs. 6% fat in liver) in salmon fed a diet where 70% of the added oil was rapeseed compared to 30% (2.8% vs. 7% EPA + DHA in feed, respectively) [14]. The authors posited that low temperature was one of the reasons for this lipid accumulation, combined with the dietary fatty acid (FA) composition. Indeed, salmon kept at 6 °C gained more fat in the liver with decreasing dietary EPA + DHA, though no such relationship was seen at 12 °C [5]. Similar results of temperature were found in Reference [7]. No clear definition of how much liver fat is normal or healthy in salmon exists as of yet, though, as described, a higher liver lipid content seemingly cooccurs with reduced robustness. 

High dietary EPA and DHA is known to affect hepatic lipid metabolism in mammals, resulting in decreased liver fat with higher dietary levels of these FA [15]. However, the lipid-lowering effects of EPA and DHA appear to differ from one another [16,17]. For salmon, dietary EPA + DHA have been proposed to inhibit lipogenesis when sufficiently high [18,19] and increase FA oxidation [20,21,22]. Incubating salmon hepatocytes with EPA resulted in an increased mitochondrial area and a reduced secretion of glycerolipids, suggestive of higher mitochondrial β-oxidation [23]. EPA has also been reported to increase the number of hepatic mitochondria [24]. Salmon fed diets with high DHA and very low EPA (algal oil) had a higher occurrence of pale livers and a higher total lipid level in the liver than fish fed FO. The authors hypothesised the reason to be mild chronic stress in the liver caused by a lack of EPA [25]. DHA increases the peroxisomal β-oxidation capacity in salmon [26]. The peroxisomal β-oxidation capacity in salmon liver is high [27] and can account for a considerable amount of the hepatic β-oxidation [20]. In contrast to the aforementioned results, few or no effects on the hepatic β-oxidation capability were seen in salmon fed diets with 100% of the added oil being vegetable oil (rapeseed oil or a vegetable oil blend) compared to fish oil during the seawater phase [28]. Exchanging FO with VO blends (rapeseed, linseed and palm oil) was thought to cause fat accumulation in the liver by inhibiting the production of very low-density lipoproteins (VLDL), which reduced the secretion of lipids and, hence, caused lipid deposition [29]. Thus, EPA and DHA have several ways of influencing hepatic lipid storage. 

The exact mechanisms behind hepatic fat accumulation remains unclear, as there are many pathways, with different regulatory steps, that have been proposed to contribute to this phenotype. As mentioned, there can be an increase in lipogenesis, the inhibition of FA oxidation, increased triacylglycerol (TAG) synthesis, an altered uptake of FA into the liver or a decreased clearance of lipids from the liver due to an imbalanced FA composition. However, other dietary components or environmental factors can also have an effect. The use of metabolomics approaches in fish nutrition is a relatively recent field and provides an unbiased investigation of the effects of the feed on the metabolism [30,31]. Nutrigenomics, namely the investigation of the relationship between nutrients and gene expression, has provided valuable knowledge in the field of fish nutrition. However, it is limited by not studying downstream post-translational effects or protein activities [30]. Metabolomics focuses on the metabolites present in the sample, hence giving an overview of the metabolic activities at a given time point [30], greatly aiding in explaining the observed phenotype. 

While 10 g of EPA + DHA per kg feed is considered too low for salmon [2], with significantly higher mortality rates after delousing compared to higher levels, an increase in dietary EPA + DHA from 16 to 26 g/kg yielded no differences in robustness [32]. Despite the fish in this study going through multiple lice treatments and facing significant disease outbreaks of, i.e., pancreas disease and gill infections, no significant difference in mortality was observed. Hence, the current requirements of EPA and DHA for salmon are thought to be between 10 and 16 g per kg of formulated feed (for commercial growing out diets with 35–39% lipids). Currently, Norwegian salmon feed contains, on average, 24 g of EPA + DHA per kg feed (surveillance of commercial Norwegian salmon feeds [33]); the lowest dietary level found was 16.6 g/kg. This is above the expected minimum requirement of salmon at 10–16 g/kg. However, due to the limited global supply of FO and fishmeal, the pressure for reduced inclusion is ever increasing. Therefore, the present study investigated dietary EPA + DHA at 10, 13, 16 and 35 g/kg (corresponding to 2.8, 3.8, 4.7 and 10.2% of the total FA (TFA), respectively) fed to Atlantic salmon during a full growing out phase in sea cages. The final diet at 35 g/kg was included to reveal whether beneficial effects of EPA + DHA higher than the current commercial levels could be seen. The goal of this paper was to investigate the relationship between hepatic lipid metabolism and EPA + DHA in the feed by using an untargeted metabolomics approach. 

## 2. Results

### 2.1. Dietary Fatty Acids and Proximate Composition

The dietary proximate and FA composition are presented in Table 1. The diets will be referred to by their planned percentage of EPA + DHA in the diet (Diet 1.0, diet 1.3, diet 1.6 and diet 3.5). All diets were isoproteic (~33%), isolipidic (~38%) and isoenergetic (~26 MJ/kg). Dietary EPA + DHA increased nicely across the diets, as planned. Concurrently, reductions in 18:1n-9, 18:2n-6 and 18:3n-3 and a slight increase in saturated FA (SFA) were seen.

### 2.2. Growth Is Higher and Hepatosomatic Index Is Lower with Higher Dietary EPA + DHA

The average fish size at the final sampling was roughly 5 kg; however, fish that had been fed diet 3.5 had significantly higher final weights, a specific growth rate (SGR) and thermal growth coefficient (TGC) compared to fish fed the other diets (Table 2). The feed intake was similar in all diet groups. Further details on the growth performance, mortality, welfare scores and fillet quality were thoroughly discussed in Lutfi et al. [34]. The hepatosomatic index (HSI) decreased significantly with increasing dietary EPA and DHA (Table 2). 

### 2.3. Lipid Classes—Significantly More Fat in the Livers of Fish Given Diet 1.0 or 1.3 than Fish Given Diet 3.5

**Figure 1:**
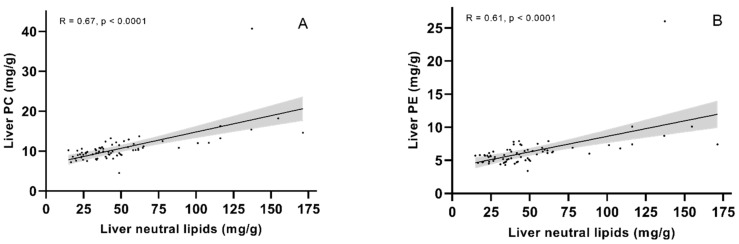
Correlations between sum neutral lipids and (**A**) phosphatidylcholine (PC) or (**B**) phosphatidylethanolamine (PE) in the liver of Atlantic salmon fed diets with increasing dietary EPA + DHA. Spearman’s rank correlation coefficient.

The fish fed diet 1.3 had significantly higher levels of phosphatidylethanolamine (PE) and phosphatidylcholine (PC) in the liver than fish fed diet 3.5. The same dietary groups also showed a significantly higher sum of liver polar lipids (Table 3). The mean levels of PE, PC and sum polar lipids were numerically high in the 1.0 diet group but not statistically different from the other groups due to larger individual variations. In all dietary groups, there was an unusually high content of free fatty acid (FFA) combined with less TAG than expected for fish of this size (data not shown). This likely indicates thawing of the samples at some point, allowing the release of FFA from TAG [35]. Hence, data for the individual neutral lipid classes are not provided. However, the results on the sum of neutral lipids are still valid. The fish fed diet 3.5 had significantly lower sum neutral lipids than the other diet groups (Table 3). The standard deviations were large in fish fed diets 1.0 and 1.3, where almost all outliers were found. There was a significant correlation between PC and PE with sum neutral lipids (Figure 1). When analysing within the dietary groups, the correlations were still significant within diet group 1.0 (R = 0.76, *p* = 0.0002 and R = 0.75, *p* = 0.0004 for PC and PE, respectively) and 1.3 (R = 0.74, *p* = 0.0005 and R = 0.5, *p* = 0.034 for PC and PE, respectively).

### 2.4. FA Composition in Neutral and Polar Lipids—Neutral Lipids Accumulate Oleic Acid When Dietary EPA + DHA Is Low

The FA composition of the liver polar lipids is presented in % of TFA (Table 4). EPA + DHA increased significantly in liver polar lipids with a dietary content. EPA + DHA was higher in polar lipids than in the feed. The sum of n-6 FA decreased with increasing EPA + DHA in the feed, concurrent with the dietary n-6 content. However, arachidonic acid (ARA, 20:4n-6) showed the opposite direction. The SFA were stable in diet groups 1.0, 1.3 and 1.6, while fish fed diet 3.5 had significantly more than the other diet groups. SFA were also higher in the polar lipids than in the feed. Monounsaturated FA (MUFA) decreased significantly with more dietary EPA + DHA, concurrent with the dietary content of the MUFA oleic acid. 

Full FA acid composition of polar lipids of the liver in mg/g and % of TFA are presented in Table A1 and Table A2, respectively.

The FA composition of the liver neutral lipids is presented in mg/g (Table 5) due to high individual variations of total fat. However, for comparisons of diets, the FA profile is used. Although EPA + DHA increased in the liver neutral lipids with the dietary content, the amount present is lower than in the feed. The sum n-6 FA was reduced with increasing dietary EPA + DHA/decreasing sum n-6; however, the differences between the dietary groups were smaller than the differences in feed. There were higher amounts of oleic acid in liver neutral lipids with less dietary EPA + DHA, which was the same pattern as seen for oleic acid in the feed. The oleic acid content of the liver neutral lipids was higher than in the feed. SFA decreased significantly with the increasing dietary EPA + DHA and was present in lower quantities than in the diet. As in the lipid class data, the standard deviations in TFA of the neutral lipids were considerably larger in the 1.0 and 1.3 diet groups compared to the two other dietary groups. 

The full FA composition of the neutral liver in mg/g and % of TFA are presented in Table A1 and Table A2, respectively.

### 2.5. Metabolomics

#### 2.5.1. Differences between the Groups Based on Overall Metabolite Signature

In the current metabolomics dataset, 795 biochemicals of known identity were detected. The biggest difference was observed between diet groups 1.0 vs. 3.5 (241 differentially regulated compounds), followed by diet 1.6 vs. diet 3.5 (199 differentially regulated compounds) and, finally, diet 1.0 vs. diet 1.6 (102 differentially regulated compounds). These results show that the dietary level of EPA + DHA affects the liver metabolic profile of salmon. Furthermore, they demonstrate that the metabolite profile of fish fed diet 10 and 16 are more similar, while the fish fed diet 3.5 is more distinct from the other two. 

**Figure 2:**
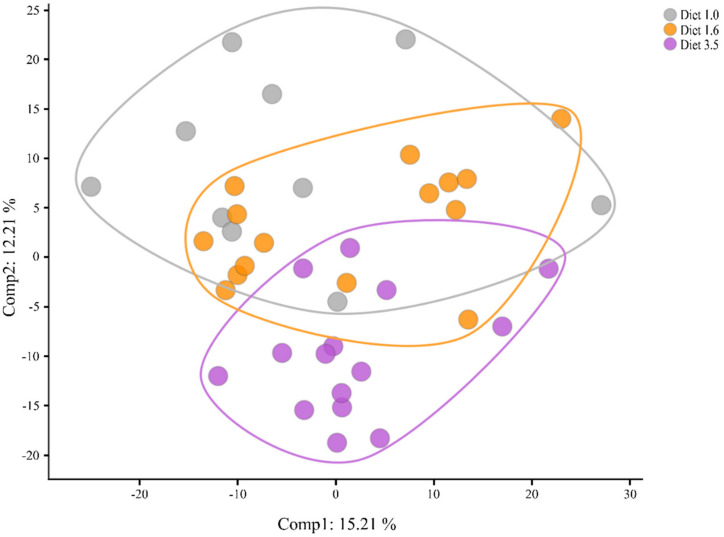
PCA of liver samples from Atlantic salmon fed diets with increasing dietary EPA + DHA: diet 1.0 (grey), diet 1.6 (orange) and diet 3.5 (purple).

As PCA allows for the visualisation of individuals within a group based on their data-compressed principal components, it can also help in determining whether the dietary groups differ from one another based on their overall metabolite signature. As illustrated in Figure 2, PC1 vs. PC2 show that fish fed diets 1.0 and 1.6 are intermixed, whereas fish fed diet 3.5 separate more from the other two. They separate based on PC2, which explains 12.3% of the variation. Hence, a phenotypic difference in the metabolome originating from the dietary EPA + DHA is evident.

#### 2.5.2. Core Findings in the Metabolomics Data

The metabolomics data indicate that feeding salmon lower levels of EPA and DHA leads to an impaired energy production in the liver (overview in Table 6). Notably, it appears to compromise β-oxidation, potentially through the carnitine shuttle for transport of FA into the mitochondria or the β-oxidation reactions. This is in line with the results in other pathways that seemingly increased their activity to compensate, such as branched chain amino acid (BCAA) catabolism and the pentose phosphate pathway (PPP). The phospholipid (PL) metabolism and lysophospholipid homeostasis also appear out of balance. The full metabolomics dataset can be found in the Supplementary Materials.

#### 2.5.3. The TCA Cycle, Pentose Phosphate Pathway, Creatine Metabolism and BCAA Metabolism Have Altered Activity When Decreasing the Dietary Content of EPA + DHA

Decreasing the dietary content of EPA and DHA resulted in changes in the metabolites involved in the tricarboxylic acid (TCA) cycle, the PPP, creatine metabolism and the BCAA metabolism (Table 7). These are all metabolic pathways that can be used for energy production. Citrate, aconitate and isocitrate were all reduced in the liver of fish given diet 1.0 compared to fish given diets 1.6 and 3.5 (Table 7). The difference was not significant between diets 1.0 and 3.5 for citrate and aconitate, possibly due to the fewer replicates in diet 1.0. Still, this result indicates a reduced input of β-oxidation products into the TCA cycle in fish fed diet 1.0 compared to the other two diet groups. Further, acetyl-CoA was numerically lower in diet groups 1.0 and 1.6 than in diet group 3.5. In the PPP, ribulose-5-phosphate was significantly increased in fish given diet 1.0 compared to the other diets. Galactonate, which feeds into the PPP, was also increased in fish given diets 1.0 and 1.6 compared to fish fed diet 3.5. This could imply an increased activity of the PPP to supply energy in the fish fed the diets with the lowest EPA and DHA contents. 

**Figure 3:**
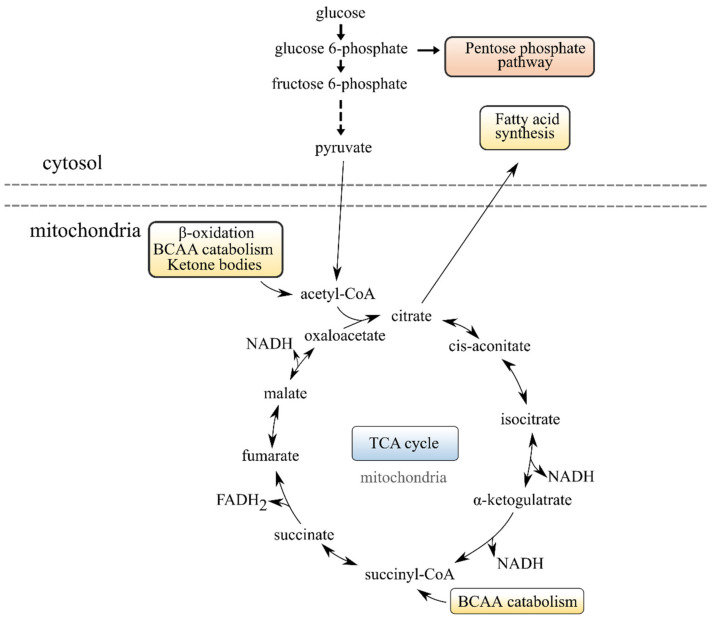
Illustration of how the tricarboxylic acid (TCA) cycle, pentose phosphate pathway, branched chain amino acid (BCAA) catabolism, fatty acid (FA) synthesis and β-oxidation are linked together in the cell.

Several metabolites in the BCAA metabolism were significantly higher in fish given diet 1.0 compared to fish given the diets with higher EPA + DHA contents (Table 7). This included the BCAA themselves, dipeptides containing BCAA (incomplete protein degradation products), propionylcarnitine (from isoleucine and valine catabolism) and butyrylcarnitine (from leucine and isoleucine catabolism) [36]. Some metabolites of BCAA metabolism were also significantly higher in fish fed diet 1.6 compared to diet 3.5. The BCAA are likely an alternative to FA for introducing intermediates into the TCA cycle via succinyl CoA (Figure 3) in fish fed the two lowest EPA and DHA levels investigated here. It is therefore fitting that succinate was numerically higher in fish fed diets 1.0 and 1.6 compared to fish fed diet 3.5 (Table 7). In creatine metabolism, creatine and creatinine are increased or trending higher in fish fed diets 1.0 and 1.6, while guanidinoacetate is numerically higher (Table 7). Together, these results indicate a perturbation of the energy homeostasis, possibly related to the changes in the TCA cycle, PPP and BCAA catabolism, particularly in fish fed diet 1.0. 

See Figure 3 for an illustration of how the TCA cycle, PPP and BCAA catabolism interconnect. 

#### 2.5.4. An increased Occurrence of Free Carnitine and Acyl Carnitines in the Lower EPA + DHA Dietary Groups Point to Issues with the Mitochondrial β-Oxidation Capacity

**Figure 4:**
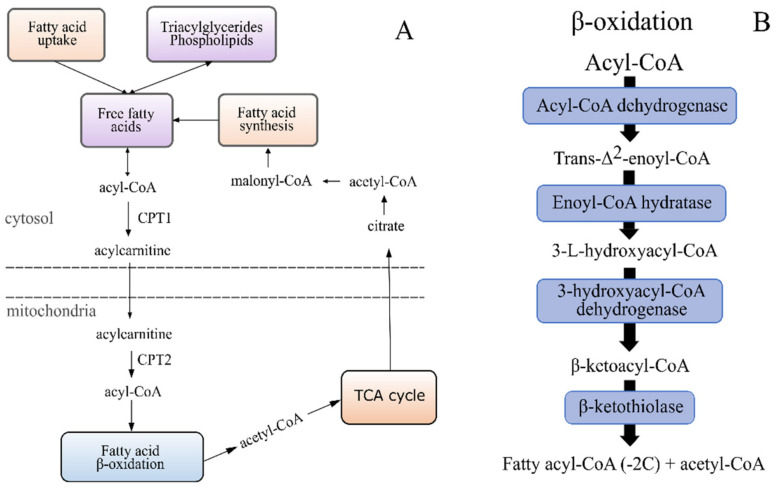
Illustration of the cycle of FA transport into the mitochondria, β-oxidation, influx of FA oxidation products into the TCA cycle and transport of citrate out of the mitochondria for FA synthesis (**A**). The β-oxidation reactions (**B**).

There was significantly more free carnitine in fish fed diets 1.0 and 1.6 compared to fish fed diet 3.5. Deoxycarnitine (carnitine precursor) was also higher in these two groups, although only significant for diet 1.6 vs. diet 3.5 (Table 8). Furthermore, compared to fish given diet 3.5, fish fed diet 1.6 had significantly more acylcarnitines of various FA. Several acyl carnitines were also higher in diet 1.0 compared to diet 3.5 (Table A3), though there were fewer statistically significant differences. This could be due to a weaker statistical strength with fewer replicates in diet group 1.0 (see Section 4.5). However, the 3-hydroxyacylcarnitines were significantly higher in fish fed diets 1.0 and 1.6 compared to fish fed diet 3.5 (3-hydroxyacyl CoA are intermediates in β-oxidation; Figure 4) (Table A3). These results are indicative of an ineffective mitochondrial β-oxidation, due to either a lower transport of FA into the mitochondria, inability to reform acyl-CoA within the mitochondria or an issue with the mitochondrial β-oxidation reactions when fish are fed diets 1.0 and 1.6 compared to diet 3.5. 

See Figure 4 and how it illustrates the link between FFA in the cytosol and β-oxidation in the mitochondria (transport via acyl carnitines) with its link to the TCA cycle. 

#### 2.5.5. A Lower Dietary EPA + DHA Leads to Altered Phospholipid Metabolism, with Higher Levels of Phospholipid Degradants and Lysophospholipids

Evidence of an altered PL metabolism in the two dietary groups with the lowest EPA + DHA is seen by the increased levels of glycerophosphoethanolamine, glycerophosphoserine and glycerophosphoinositol compared to the highest EPA + DHA group. These are degradants of phosphatidylethanolamine, phosphatidylserine and phosphatidylinositol, respectively. Furthermore, the two lower EPA + DHA groups have a higher content of almost all lysophospholipids compared to the high group. Fish fed diet 1.0 also showed significantly higher levels of many of these lysophospholipids than fish fed diet 1.6 (Table A4). 

#### 2.5.6. Alterations in Eicosanoid and Tryptophan Metabolites Levels When Changing Dietary EPA and DHA 

The 5-lipoxygenase product 5-HEPE, an EPA-derived eicosanoid, was significantly higher in the fish fed diet 3.5 compared to fish fed diets 1.0 and 1.6. Fish fed the two lower EPA + DHA diets had significantly more kynurenine than fish fed the high diet, and fish fed diet 1.6 also had significantly more kynurenate than fish fed diet 3.5 (Table 9). Higher levels of kynurenine in fish fed diet 1.0 and diet 1.6 can reflect the increasing levels of inflammation compared to fish fed diet 3.5. 

#### 2.5.7. Gene Expression

**Figure A1:**
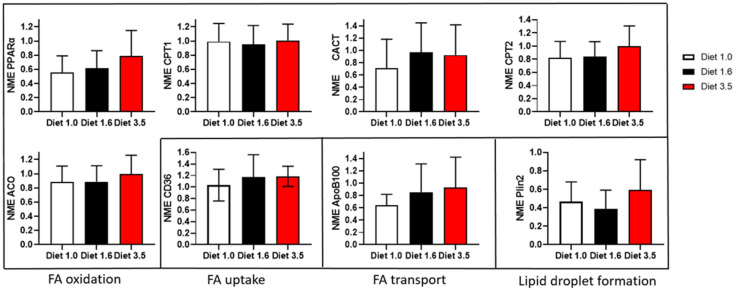
Normalised mean expression (NME) of selected genes involved in fatty acid (FA) usage in the liver of Atlantic salmon fed with increasing dietary EPA + DHA contents. Triplicate cages per diet group and five fish sampled from each cage (*n* = 15). Analysed with one-way ANOVA with Tukey’s HSD post hoc.

There were no significant differences between the dietary groups in the gene expression of *ppara*, *cpt1*, *cact*, *cpt2*, *aco* (FA oxidation), *cd36* (FA uptake), *apob100* (FA transport) or *plin2* (lipid droplet formation) (Figure A1).

## 3. Discussion

Hepatic lipid accumulation is considered a general sign of dietary imbalance and indicates a perturbation in energy metabolism [37]. Lowering the dietary content of EPA + DHA (≤10 g/kg) in salmon feeds has been associated with increases in the hepatic lipid level [2]. Data from the present study (HSI, lipid class and mg/g total FA) clearly illustrate an increase in liver lipids with lower dietary EPA + DHA intakes. Livers of fish fed diets 1.0, 1.3 and 1.6 had fat contents around 7–12%, while, in livers of fish fed diet 3.5, they only had 5% fat. Other trials with comparable dietary EPA + DHA to the three lowest dietary EPA + DHA of the present trial, and temperatures at ~6 °C have reported liver fat contents ranging from 7 to 17% [5,7,38]. Salmon provided >16-g EPA + DHA per kg feed are reported with liver fat levels around 6 to 7% [7,14,32], similar to this study reporting 5% liver fat I diet group 3.5. Although there is no clear threshold indicating a detrimental level of liver fat, dietary EPA + DHA above 16 g/kg results in lower, more stable liver fat contents. The variation in the lipid data within diet groups 1.0 and 1.3 was large, with some individual fish having markedly higher lipid contents than other fish fed the same diet. Liver neutral lipids from the lipid class data correlated well with both PC and PE for fish fed the two diets lowest in EPA + DHA, similar to the results of Jordal et al. [29]. Both PC and PE are needed for the formation of membranes surrounding lipid droplets. This could indicate that fish fed the lower levels of EPA + DHA in the present study formed more lipid droplets in the liver, similar to previous observations in salmon fed low EPA + DHA [3]. In accordance with this, a tendency of a decreased abundance of lipid droplets was found in the fish fed the highest dietary level of EPA + DHA in their livers in the present study [34]. Liver colour is regarded as a visual indicator of liver lipids [39], with a paler liver indicating higher fat contents and possible nutritional disorders [40]. The fish fed diet 3.5 further had marked improvement in liver colour compared to the low EPA + DHA diets [34]. Their colour was better in fish fed diet 1.6 compared to 1.0 and 1.3 but was drastically improved in fish fed diet 3.5 compared to the other diet groups. This further supports a lower lipid accumulation in diet group 3.5. Notably, there were very few outliers in diet group 1.6 and none in diet group 3.5 in the liver lipid class and FA composition data. The individual capabilities of the fish are likely crucial to the fish’s ability to handle a low EPA + DHA diet, and the dietary supply must be enough to ensure the requirements of all fish are covered. Oleic acid was the major FA in the accumulated neutral fat (39.4–52.9% of TFA in neutral lipids), being higher in the neutral lipids than in the feed. This indicates that oleic acid accumulates at the expense of other FA, and it has been suggested that it is metabolised slower than other FA in salmonids [41]. Furthermore, oleic acid-rich diets stimulate hepatic TAG accumulation in salmon to a greater degree than EPA- and DHA-rich diets [24], and higher levels of added oleic acid to salmon hepatocytes resulted in increased lipid droplet formation [42]. In HepG2 cells, oleic acid led to TAG accumulation, larger lipid droplets and a closer proximity between lipid droplets and mitochondria than when cells were treated with bovine serum albumin (control) or palmitic acid. Additionally, mitochondria from the cells added oleic acid and reduced the FA oxidation capacity compared to the control or palmitic acid-treated cells [43]. These results match well with observations from the present study and imply that increased oleic acid, which was elevated when EPA + DHA decreased, can contribute to lipid accumulation in the liver. 

To further consider the possible mechanisms causing these increases in the liver lipids in our study, a metabolomic profiling of the liver was performed. The great advantage of metabolomics is its provision of a large suite of unbiased data, which can then be used to deduce the biological processes behind the observed changes [30,31]. In the present study, the global overview of the data (PCA) indicated that the metabolite signature of the fish separated based on the EPA + DHA content of their feed. Fish given diets 1.0 and 1.6 clustered together, while fish fed diet 3.5 separated slightly from the other two groups. The metabolic profile was suggestive of a disturbed energy homeostasis, with the fish seemingly unable to fully use lipids and FA for energy production when fed lower levels of EPA + DHA. 

We found a significant increase in the hepatic lysophospholipid levels in fish fed diets 1.0 and 1.6 compared to diet 3.5, which, in mammals, has been found to suppress FA oxidation in the liver [44,45] by altering the mitochondrial membrane permeability [44]. Crossing the mitochondrial membrane is the first step of mitochondrial β-oxidation and is catalysed by CPT1 (formation of acylcarnitine from acyl-CoA), carnitine acyl-carnitine translocase (CACT, facilitates transport) and CPT2 (reformation of acyl-CoA within mitochondria) [46]. An increase in hepatic acylcarnitine in fish fed diets 1.0 and 1.6 compared to diet 3.5 was concurrent with the elevated lysophospholipid levels in the present study, indicating an issue with FA oxidation. A disturbance of the mitochondrial membrane causing an issue with the efficiency of CACT or CPT2 could be a possible explanation. Increased activity and gene expression of CACT have been found in rat livers [47] and increased gene expression in the hepatopancreas of grass carps [48] with higher dietary EPA + DHA. However, we did not find any significant differences between the diet groups in the mRNA levels of CACT. Similarly, studies have shown an increased gene expression of CPT2 in salmon livers [22] and CPT2 activity in brown trout livers [49] with more EPA + DHA in the feed, but no such differences were seen in our gene expression data. Hence, the issue is possibly not with the transport of FA into the mitochondria. However, a significantly higher accumulation of 3-hydroxyacylcarnitines was evident in fish fed diets 1.0 and 1.6 compared to diet 3.5 in the current study, which indicates dysfunction with 3-hydroxy-acyl-CoA-dehydrogenase (third step in mitochondrial β-oxidation) [50]. A disturbance of the mitochondrial membrane, suggested to have occurred in fish fed low EPA + DHA in the present study, could cause problems for this enzyme, as it is located on the mitochondrial membrane [51]. A general accumulation of acylcarnitines would be expected concomitant with hydroxylated acylcarnitines; however, the accumulation was less pronounced in fish fed diet 1.0 than diet 1.6. Nonetheless, the metabolic profile of the fish fed diet 1.0 distinctly demonstrated a shift away from using FA as energy. An incomplete β-oxidation cycle would lead to a deficit in acetyl-CoA for the TCA cycle. The numericreduction in acetyl-CoA, combined with reduced citrate, aconitate and iso-citrate in fish diet 1.0, support this. Although some metabolites were only numerically different, they all pointed in the same direction as other statistically significant metabolites. Altogether, this could result in the observed phenotype of accumulating fat in the livers of salmon fed less dietary EPA + DHA.

Studies on the effects of EPA + DHA in the feed on β-oxidation in salmon generally support that it increases with a higher dietary content of these FA [20,21,23,24,52], though some studies have reported no effects (≤19.3% EPA + DHA of TFA) [28,53]. The current metabolomics data suggests that a reduced EPA + DHA in the liver membranes might hamper lipid usage through altered membrane functionality. This is in line with the results of Závorka et al. [54], who found that juvenile wild salmon fed less EPA + DHA had reduced mitochondrial efficiency in the muscle. An efficient mitochondrial activity is closely related to the growth performance in salmon [54] and brown trout [55]. In accordance with this, we found a significantly higher growth and a better functioning lipid metabolism in salmon fed the highest level of EPA + DHA. Although pathological lipidosis in the liver was not observed, the lower EPA + DHA diet groups had significantly more fat in their livers. Higher mortality rates tend to coincide with an increased frequency of fatty liver [13,14]. The mortality in these studies was suggested to be induced by a combination of low temperatures (~5 °C) and lipid nutrition (switching to a diet higher in lipids [13] or high dietary oleic acid [14]). There was also a tendency of reduced mortality caused by a single, natural outbreak of cardiomyopathy syndrome (CMS) (confirmed by a veterinarian) with increasing dietary EPA + DHA levels in the present study (reported by Lutfi et al. [34]). While this does not necessarily imply that elevated liver lipids in itself cause increased mortality, it is, at the very least, a symptom of a less robust fish. Studies have found that salmon reared at 6 °C will gain more liver fat than fish reared at 12 °C, even when provided the same dietary EPA + DHA level, demonstrating that the response to dietary EPA + DHA is influenced by temperature [5]. Insufficient dietary EPA + DHA to maintain normal membrane functionality, particularly at low temperatures, could thus result in reduced mitochondrial efficiency, reduced growth, increased liver lipids and, ultimately, a salmon less robust when exposed to additional challenges. 

Changes to the dietary n-3 and n-6 FA contents in salmon feeds result in altered eicosanoid production [56,57], and we have previously linked hepatic increases in prostaglandin E_2_ and prostaglandin D_2_ to elevated liver TAG [56]. In the present study, the metabolomics data revealed a decrease in the EPA-derived eicosanoid 5-HEPE when lowering the dietary EPA + DHA. Wang et al. [58] discovered that 17,18-EEQ, 5-HEPE and 9-HEPE (EPA metabolites) ameliorated NAFLD induced by a high fat diet in mice. Their study indicated that these eicosanoids attenuated the inflammatory response of macrophages in adipose tissue, which further ameliorated liver lipid accumulation. As reviewed in Duan et al. [59], there are several metabolites of EPA, but also DHA, that have been demonstrated to improve hepatic steatosis in mammals. Concurrent with the reduced 5-HEPE in the livers of fish fed diets 1.0 and 1.6, a significant increase in lysophospholipids was found. The secretion of adiponectin by adipose tissue is reduced in obesity (see Reference [60]). This adipokine directly reduces the hepatic lysophospholipid levels in mammals [61]. Hence, liver lysophospholipids are increased in obesity. These data support the interplay between adipose and hepatic tissue in the development of a fatty liver-like phenotype. EPA and DHA increased the rates of β-oxidation and reduced TAG in salmon adipose tissue [62] and cultured adipocytes [63], and DHA has been demonstrated to protect salmon adipocytes against inflammation [64]. This possible connection between an inflamed adipose tissue and fatty liver-like phenotype in salmon is also something that deserves further attention. 

Long-chain acylcarnitines can meditate proinflammatory responses in cultured immune cells [65], and they can prompt an expression of proinflammatory cytokines following a dose-dependent manner [66]. In addition to the accumulating acylcarnitines in the 1.0 and 1.6 diet groups, higher levels of kynurenine and kynurenate were observed. Tryptophan 2,3-dioxygenase (TDO, functioning in the liver) and indoleamine 2,3-dioxygenase (IDO, extrahepatic) are two enzymes that catalyse the first step of the kynurenic pathway, and they are both induced by inflammatory cytokines (reviewed in Reference [67]). These findings support a higher state of inflammation in fish fed these diets, possibly partly due to the accumulating acylcarnitines resulting in increased proinflammatory cytokines. Kynurenine and kynurenic acids, in opposition to other kynurenine metabolites, are anti-inflammatory (reviewed in Reference [68]) and can serve as a brake on the immune response. Hence, although they are a sign of a higher inflammatory state, they can help alleviate inflammation. 

Salmon are exposed to many challenges under farming conditions, including diseases and handling, which can lead to substantial mortalities and suboptimal performance. Whether the fishes’ robustness is affected by dietary levels of EPA + DHA is of great importance, as it is an issue both of animal welfare and economic interest. This study clearly demonstrated that low dietary EPA + DHA (≤13 g/kg, diet 1.0 and 1.3) can cause elevated liver lipids and a fatty liver-like phenotype in the metabolite profile, similar to many published studies showing higher liver lipids with decreasing dietary EPA + DHA in salmon [2,3,4]. We found indications of a higher inflammatory status in fish fed diets 1.0 and 1.6 than in fish fed diet 3.5. Similarly, other studies have shown that a reduced dietary inclusion of EPA + DHA can result in an increased inflammatory response [69,70,71,72,73]. EPA + DHA at the highest level (diet 3.5) greatly ameliorated all metabolic health issues investigated in the present trial; these fish did not have hepatic lipid accumulation, had a normally functioning lipid metabolism, reduced inflammatory metabolite markers and they achieved significantly higher final weights. This is concurrent with what we observed in the metabolic profile, where fish fed diets 1.0 and 1.6 grouped together and diet group 3.5 separated slightly from the other two. Combined, these results indicated a more robust fish when fed the highest EPA + DHA levels. The substantial variation seen only in the data from diet groups 1.0 and 1.3 indicate that, while some individual fish may cope with low dietary levels of EPA + DHA, many will have reduced robustness. However, to ensure the robustness of all fish and avoid losses, it is important that sufficient levels of EPA + DHA be included in diets.

The current study indicated a dysregulation of the hepatic energy metabolism of Atlantic salmon when they were fed lower levels of EPA and DHA. Through a range of different mechanisms, the ability to use lipids for energy was reduced, and the fish needed to initiate alternative strategies for energy production. Feeding diet 1.0 was demonstrated to provide insufficient EPA + DHA for a normally functioning liver lipid metabolism. Although fish fed diet 1.6 showed some improvement compared to fish fed diet 1.0, an even further improvement was seen when going from diet 1.6 to diet 3.5. This was indicative of diet 1.6 also being suboptimal for maintaining a healthy liver metabolism. The difference in dietary EPA + DHA in diets 1.6 and 3.5 was relatively large, and further studies are therefore required to find the optimal dietary inclusion of EPA + DHA within this range for a healthy hepatic lipid metabolism. 

## 4. Materials and Methods

### 4.1. Diets and Fish Trial

This trial, its experimental design and fish performances have been described in detail elsewhere [34]. Briefly, four diets with increasing levels of EPA + DHA were formulated (10, 13, 16 and 35 g of EPA + DHA/kg feed, corresponding to 2.8, 3.8, 4.7 and 10.2% of TFA, respectively). The EPA:DHA ratios were approximately 1:1 in all diets. Fish meal was included at 5% of the recipe in all diets of the two final feed batches, which are the most relevant here. To modulate the EPA + DHA content, FO replaced rapeseed oil in the diets. All diets were formulated to be isoproteic, isolipidic and isoenergetic. In order to meet the specific dietary requirements of the fish throughout the experiment, three pellet sizes (4, 6 and 9 mm) and five feed batches were produced. Dietary proximate and FA composition (from the final feed batch) are given in Table 1. More detailed information on feed formulation for all batches, proximate and FA composition for the other batches can be found by Lutfi et al. [34]. 

Atlantic salmon post smolts (average weight 115 g) were purchased from Mowi AS (formerly Marine Harvest Norway AS. Glomfjord, Meløy, Norway). They were acclimated to the environmental conditions at Gildeskål Research Station (GIFAS, Inndyr, Norway) and fed a commercial diet for this size (BioMar AS, Trondheim, Norway) in advance of the trial. At the start of the trial, fish (average weight 275 g) of both sexes were haphazardly distributed into sea cages (5 × 5 × 5 m–125 m^3^) with 190 individuals per cage (triplicate cages per diet). The feeding trial began in October 2017 and lasted until January 2019. The fish were fed to apparent satiation once a day during the autumn and winter and twice a day during the spring and summer. Temperature (measured at 1, 3 and 5 m in depth); salinity and oxygen (measured at a 3-m depth) were monitored daily. Average temperature overall was 7 °C, declining to 3 °C during the winter and rising to 16 °C during the summer. In September 2018, an outbreak of cardiomyopathy syndrome was diagnosed. However, few fish were found to have developed severe symptoms, and all sampled fish appeared healthy. This disease outbreak is therefore not expected to have had much of an effect on the metabolomics analysis. A CMS scoring assessment was performed by an experienced histopathologist (Dr. Øystein Evensen) from the faculty of Veterinary Medicine (Norwegian University of Life sciences, Oslo, Norway). All dead fish were analysed specifically for the CMS outbreak to assess potential differences in fish robustness due to dietary differences. Mortality was recorded throughout the experiment. The trial was conducted according to the National Guidelines for Animal Care and Welfare published by the Norwegian Ministry of Education and Research (Norwegian Food Safety Authority (FOTS), approval no. 16059).

### 4.2. Sampling

At the beginning of the trial (October 2017), the fish were individually weighed and lengths measured, and they were haphazardly distributed into the experimental sea cages. At the end of the trial (January 2019), a final sampling was performed. Fish were haphazardly collected and sacrificed by an anaesthetic overdose (Tricaine Pharmaq, PHARMAQ AS, Norway, 0.3 g/L). All fish were weighed and measured individually, and livers were excised and weighed. The liver was cut into two pieces in the middle, and whole vertical slices were sampled from the middle part. Pieces of liver were then taken for an analysis of lipid class (6 fish per cage), FA composition (4 fish per cage), gene expression (5 fish per cage) and metabolomics (5 fish per cage) and flash-frozen in liquid nitrogen before storage at −80 °C until analysis. Samples for each analysis were taken from the same part of the liver each time. Fish were fed until the last day before sampling. 

### 4.3. Lipid Analyses

#### 4.3.1. Lipid Class

Lipid classes were separated by double-development high-performance thin-layer chromatography (HPTLC) using 10 × 10-cm plates (VWR, Lutterworth, UK) based on the methods reported by Henderson and Tocher et al. [74]. Samples of the total lipids (1 to 2 μg) were applied to the plate origins and the plates resolved in a methyl acetate/isopropanol/chloroform/methanol/0.25% KCl (25:25:25:10:9, *v*/*v*) aqueous phase to 5.2 cm. The plates were then removed from the aqueous phase, the excess solvent evaporated via air drying and vacuum desiccation and the plates then further developed to 9.5 cm using a second solvent mixture containing isohexane/diethyl ether/acetic acid (80:20:1, *v*/*v*) before termination and drying, as detailed previously. The different lipid classes were visualised following spraying the plates with a 3% (*w*/*v*) cupric acetate solution containing 8% (*v*/*v*) phosphoric acid; after which, the plates were charred at 160 °C for 20 min. Lipid classes were quantified by densitometry using a CAMAG-3 TLC Scanner (version Firmware 1.14.16; CAMAG, Muttenz, Switzerland) using winCATS software (Planar Chromatography Manager, version 1.2.3).

#### 4.3.2. Fatty Acid Composition

Total lipids were extracted from liver samples with chloroform/methanol (2:1, Merck, Darmstadt, Germany) with roughly 20 times the sample weight and frozen overnight at −20 °C. Neutral and polar lipids of the sample extracts were separated by solid phase extraction before analysis of the FA composition, as described by Sissener et al. [75]. Nonadecanoic acid (19:0) was added to all samples as an internal standard for quantitative determination. Briefly, extracts were filtered and evaporated, then saponified and methylated with BF_3_ in methanol. FA separation was conducted using the AutoGC (Autosystem XL, Perkin Elmer Inc., Waltham, MA, USA) fitted with a flame ionisation detector. For integration, Chromeleon^®^ version 7.2 (Thermo Scientific, Waltham, MA, USA) was used. 

### 4.4. Gene Expression

RNA extraction, cDNA synthesis and analysis of the gene expression was performed on liver samples, as reported in Hundal et al. [56]. Melting curves were monitored in the last amplification cycle to ensure the specificity of the primers. The absorbance ratio A260/280 was 2.1 ± 0.0, A260/230 was 2.2 ± 0.0 and the RIN-value was >7.2 in all samples, hence indicating the RNA samples acceptable for RT-qPCR. The stability of the reference genes (β-actin, ARP and EF1ab) was calculated using CFX Maestro software (Bio-Rad CFX Maestro version 1.1, Bio-Rad Laboratories, Hercules, CA, USA), which performed a stability analysis based on the GeNorm algorithm. Normalisation was performed using CFX Maestro. The PCR primer sequences can be found in Table 10. 

The primer sequence for *cact* was created from the Atlantic salmon genome using Primer 3 Software. The specificity of the new primer *cact* was tested by a QIAGEN OneStep RT-PCR Kit. First, the primer was diluted to 0.05nmol/μL with TE buffer. Then, the master mix reagent was made according to the manufacturer’s instructions. The RT reaction took place on a PCR machine (GeneAmp PCR 9700, Applied Biosystems, Waltham, MA, USA) with the following temperature program: reverse transcription for 30 min at 50 °C, PCR activation for 15 min at 95 °C, denaturation for 45 secs at 94 °C, annealing for 45 secs at 60 °C, extension for 1 min at 72 °C and a final extension for 10 min at 72 °C. The PCR product was tested on an agarose gel with Gel Red Nucleic Acid Stain as a dye by a charged voltage at 80V. After one hour, the gel was photographed by a UV light to check the results. A single band of the expected size was found in the gel.

### 4.5. Metabolomics

Analysis of metabolites was performed by Metabolon (Durham, NC, USA), as previously described [76,77], on liver samples collected from fish fed diets 1.0, 1.6 and 3.5. Due to economic constraints, only diet groups 1.0, 1.6 and 3.5 were analysed. There were 5 samples collected per cage, giving *n* = 15 for diet 1.6 and diet 3.5. Unfortunately, 5 replicates (all samples from one cage) were lost in the group fed diet 1.0, resulting in *n* = 10 in this group. For quality control purposes, several recovery standards were added prior to automated sample preparation using the MicroLab STAR^®^ system from Hamilton Company. Protein removal was performed with methanol under vigorous shaking for 2 min using Glen Mills GenoGrinder 2000 before centrifugation. The extract was fractioned and analysed by two separate reverse-phase (RP)/UPLC-MS/MS methods with positive ion mode electrospray ionization (ESI), one by RP/UPLC-MS/MS with the negative ion mode ESI and one by HILIC/UPLC-MS/MS with the negative ion mode ESI. To remove the solvent, the samples were evaporated using TurboVap^®^ (Zymark, Hopkinton, MA, USA), followed by drying under nitrogen and then reconstitution in solvent appropriate for each method. The solvents also contained several standards at fixed concentrations for injection and chromatographic consistency. A Waters ACQUITY ultra-performance liquid chromatography and a Thermo Scientific Q-Exactive high-resolution/accurate mass spectrometer interfaced with a heated electrospray ionisation (HESI-II) source and Orbitrap mass analyser operated at 35,000 mass resolution (further details in the abovementioned references). Compound identification was performed by comparison to a reference library containing retention time, mass charge ratio (*m*/*z*) and chromatographic data (including MS/MS spectral data) [78]. 

### 4.6. Calculations

Growth rates were calculated based on the average values per cage:

$$Specific\;growth\;rate\;\left(SGR\right)=\frac{\ln\;w_{1}-\;\ln\;W_{0}}{t_{1}-t_{0}}\;\times \;100\;Thermal\;growth\;coefficient\;\left(TGC\right)=\left(w_{1}^{1/3}-w_{0}^{1/3}\right)\;\times \;\frac{1000}{d^{{}^{\circ}}}\;,$$

where *W*_0_ is the start weight (g) and *W*_1_ is the final weight (g) at times *t*_0_ and *t*_1_, respectively, and *d°* is the sum of day degrees.

The hepatosomatic index (HSI) was calculated as follows

$$HSI=\frac{liver\;weight\;\left(g\right)}{body\;weight\;\left(g\right)}\;\times 100$$

### 4.7. Statistics

Statistical analyses were performed using the free software environment R (http://cran.r-project.org/, R version 3.5.3, 11 March 2019). Differences between the groups in the HSI, lipid classes, FA composition and gene expression were analysed by one-way ANOVA followed by Tukey’s HSD when significant differences were found. If cage effects were found, a nested ANOVA was used (random effect = tank). A Shapiro–Wilk test and Levene’s test were used to check the normal distribution and homogeneity of the variances in the data, respectively. Graphical evaluations for homogeneity of the variances were also conducted using a fitted vs. residuals plot and, for normality, a QQ plot. If the assumptions of normality or homogeneity were not met, a Kruskal–Wallis nonparametric test was used. Correlations to liver neutral lipids were tested for phosphatidylcholine and phosphatidylethanolamine. Statistical significance was set at *p* < 0.05. Results were provided as the mean ± standard deviation, unless otherwise stated.

In the metabolomics dataset, missing values were assumed to be below the limit of detection and imputed with the metabolites’ minimum value (deemed as informative blanks, minimum value imputation). The raw values from the samples for each metabolite was divided by the median of those samples to give a median of 1. The data were log-transformed using the natural log before statistical analysis in ArrayStudio and the free software environment R (http://cran.r-project.org/, R version 3.5.3, 11 March 2019). The metabolomics data were analysed using one-way ANOVA, followed by ANOVA contrasts. Multiple comparisons were accounted for by using the False Discovery Rate (q-value). Data were presented as fold changes between pairwise comparisons of diet groups, e.g., for the comparison diet 1.0/diet 3.5, a number below one would indicate less of the metabolite in diet group 1.0, while a number above one would indicate the opposite. Red indicates significantly higher and green significantly lower (*p* < 0.05), while pink and light green indicate *p*-values between 0.05 and 0.1

## Figures and Tables

**Table 1:** Analysed proximate and FA composition (% of TFA) of the final feed batch (9 mm, fed September 2018 to January 2019). Also published in Lutfi et al. [34].

	Dietary EPA + DHA Levels (%)			
	1.0	1.3	1.6	3.5
PROXIMATE COMPOSITION				
Moisture (%)	6.0	6.0	6.0	6.0
Energy–crude (MJ/Kg)	26.2	26.2	26.2	26.1
Protein–crude (%)	33.4	33.4	33.4	33.5
Fat–crude (%)	38.7	38.7	38.6	38.4
Ash (%)	4.4	4.4	4.4	4.4
FATTY ACIDS				
14:0	0.9	1.1	1.4	2.8
16:0	7.0	7.2	7.5	9.6
18:0	2.8	2.7	2.7	3.0
20:0	0.7	0.6	0.6	0.5
ΣSFA ^1^	12.6	13.1	13.3	17.0
16:1 n-7	1.0	1.3	1.8	3.6
18:1 n-7	2.8	3.0	3.0	3.2
18:1 n-9	46.5	45.8	43.8	33.0
20:1 n-9	1.6	1.9	2.4	4.2
20:1 n-11	0.3	0.4	0.5	1.1
22:1 n-9	0.2	0.2	0.2	0.3
22:1 n-11	0.6	0.9	1.3	3.1
24:1 n-9	0.2	0.2	0.2	0.2
Σ MUFA ^2^	53.2	53.7	53.1	48.8
16:2 n-6	0.1	0.1	0.2	0.3
18:2 n-6	20.0	18.7	16.9	12.5
18:3 n-3	8.1	8.0	7.7	5.1
20:4 n-3	0.1	0.2	0.3	0.7
20:5 n-3	1.5	2.0	2.4	5.1
22:5 n-3	0.2	0.3	0.3	0.7
22:6 n-3	1.4	1.8	2.3	5.0
EPA + DHA	2.8	3.8	4.7	10.2
ΣPUFA	31.6	31.4	30.4	30.1
Σn-3 ^3^	11.5	12.5	13.3	17.2
Σn-6 ^4^	20.2	19.0	17.2	13.1
n-6/n-3	1.8	1.5	1.3	0.8
TFA mg/g feed	354.4	356.5	357.7	362.6

^1^ Includes 15:0, 17:0, 22:0 and 24:0. ^2^ Includes 16:1 n-5, 16:1 n-9, 17:1 n-7, 18:1 n-11, 20:1 n-7. 22:1 n-7. ^3^ Includes 20:3 n-3. ^4^ Includes 18:3 n-6. DHA—docosahexaenoic acid; EPA—eicosapentaenoic acid; FA—fatty acid; MUFA—monounsaturated fatty acid; PUFA—polyunsaturated fatty acid; SFA—saturated fatty acid; TFA—total fatty acids.

**Table 2:** Growth performance and hepatosomatic index for Atlantic salmon fed diets with increasing dietary EPA + DHA. Data are shown as the mean ± SEM. Weight, SGR and TGC based on average values per cage, giving *n* = 3. HSI was calculated for 15 fish per cage, *n* = 45. Different letters within each row indicate significant differences between values determined using a one-way ANOVA with Tukey’s HSD post hoc; Kruskal–Wallis was used for HSI.

	Diet 1.0	Diet 1.3	Diet 1.6	Diet 3.5
Initial weight (g)	275.4 ± 1.4	275.2 ± 2.9	276.4 ± 0.6	277.1 ± 0.7
Final weight (g)	4748.5 ± 33.4 ^a^	4938.0 ± 85.6 ^a^	4963.6 ± 68.4 ^a^	5364.6 ± 56.9 ^b^
SGR	0.68 ± 0.0003 ^a^	0.69 ± 0.005 ^a^	0.69 ± 0.003 ^a^	0.70 ± 0.002 ^b^
TGC	3.1 ± 0.016 ^a^	3.1 ± 0.031 ^a^	3.1 ± 0.023 ^a^	3.3 ± 0.017 ^b^
HSI	1.15 ± 0.02 ^c^	1.12 ± 0.02 ^bc^	1.08 ± 0.02 ^ab^	1.05 ± 0.02 ^a^

HSI—hepatosomatic index; SGR—specific growth rate; TGC—thermal growth coefficient.

**Table 3:** Liver lipid classes (mg/g) in the liver of Atlantic salmon fed diets with increasing dietary EPA + DHA. Data are the mean ± standard deviation of three tanks per diet group with 6 fish per tank (*n* = 18). Different letters denote significant statistical differences (*p* < 0.05 one-way ANOVA with Tukey’s HSD post hoc). Numbers are the mean with standard deviation.

	Diet 1.0	Diet 1.3	Diet 1.6	Diet 3.5
PC	12.0 ± 7.6 ^ab^	11.6 ± 2.7 ^b^	10.1 ± 1.2 ^ab^	9.1 ± 1.1 ^a^
PS	1.5 ± 0.9	1.4 ± 0.3	1.5 ± 0.4	1.4 ± 0.2
PI	2.4 ± 1.5	2.2 ± 0.5	2.1 ± 0.4	2.2 ± 0.5
PA/CL/PG	1.2 ± 0.6	1.1 ± 0.4	1.3 ± 0.3	1.1 ± 0.2
PE	7.2 ± 4.9 ^ab^	6.8 ± 1.4 ^b^	5.8 ± 0.9 ^ab^	5.3 ± 0.6 ^a^
Sum polar lipids	32.3 ± 19.1 ^ab^	30.7 ± 5.9 ^b^	27.5 ± 4.2 ^ab^	24.6 ± 2.4 ^a^
Sum neutral lipids	58.4 ± 32.2 ^b^	73.2 ± 44.7 ^b^	41.2 ± 12.6 ^b^	26.9 ± 7.0 ^a^
Sum lipids	90.8 ± 48.6 ^b^	103.8 ± 48.7 ^b^	68.7 ± 15.5 ^ab^	51.9 ± 8.8 ^a^

PS—phosphatidylserine; PI—phosphatidylinositol; PA—phosphatidic acid; CL—cardiolipin; PG—phosphatidylglycerol.

**Table 4:** Liver fatty acid composition of polar lipids in Atlantic salmon fed diets with increasing dietary EPA + DHA (% of TFA). Three cages per diet group and 4 fish sampled per cage (*n* = 12). Different letters denote significant statistical differences (*p* < 0.05 one-way ANOVA with Tukey’s HSD post hoc). Numbers are the mean with standard deviation.

	Diet 1.0	Diet 1.3	Diet 1.6	Diet 3.5
16:0	10.9 ± 0.9 ^a^	11.0 ± 0.6 ^a^	11.2 ± 0.7 ^a^	12.5 ± 1.0 ^b^
18:0	6.2 ± 0.7	6.1 ± 0.4	6.1 ± 0.4	6.3 ± 0.6
Sum SFA *	18.1 ± 1.2 ^a^	18.1 ± 0.7 ^a^	18.5 ± 0.8 ^a^	20.5 ± 1.1 ^b^
16:1n-7	0.5 ± 0.1 ^a^	0.5 ± 0.1 ^a^	0.5 ± 0.0 ^a^	0.8 ± 0.1 ^b^
18:1n-9	20.9 ± 2.7 ^c^	19.4 ± 0.9 ^bc^	17.9 ± 1.0 ^ab^	12.4 ± 1.3 ^a^
18:1n-7	2.0 ± 0.2 ^a^	2.0 ± 0.1 ^a^	2.0 ± 0.1 ^a^	2.3 ± 0.1 ^b^
20:1n-9	2.2 ± 0.3 ^a^	2.1 ± 0.3 ^a^	2.3 ± 0.4 ^ab^	2.6 ± 0.6 ^b^
Sum MUFA **	26.3 ± 2.9 ^c^	24.6 ± 1.1 ^bc^	23.3 ± 1.0 ^ab^	19.3 ± 1.6 ^a^
18:2n-6	10.9 ± 1.1 ^c^	10.5 ± 0.7 ^bc^	9.2 ± 0.6 ^b^	5.3 ± 0.5 ^a^
20:2n-6	2.1 ± 0.3 ^ab^	2.3 ± 0.3 ^b^	2.3 ± 0.3 ^b^	1.8 ± 0.3 ^a^
20:3n-6	2.5 ± 0.4 ^d^	1.7 ± 0.3 ^c^	1.2 ± 0.2 ^b^	0.4 ± 0.1 ^a^
20:4n-6 (ARA)	3.1 ± 0.4 ^a^	3.1 ± 0.2 ^a^	3.3 ± 0.2 ^ab^	3.9 ± 0.3 ^b^
Sum n-6 ***	19.2 ± 0.9 ^d^	18.1 ± 0.7 ^c^	16.6 ± 0.8 ^b^	12.0 ± 0.4 ^a^
18:3n-3	2.6 ± 0.5 ^b^	2.6 ±0.3 ^b^	2.5 ± 0.3 ^b^	1.4 ± 0.4 ^a^
20:4n-3	1.3 ± 0.2 ^c^	1.2 ± 0.2 ^bc^	1.1 ± 0.1 ^b^	0.8 ± 0.1 ^a^
20:5n-3 (EPA)	8.9 ± 0.8 ^a^	9.5 ± 0.6 ^ab^	10.1 ± 0.6 ^b^	12.0 ± 0.7 ^c^
22:5n-3	2.8 ± 0.3 ^a^	2.8 ± 0.2 ^a^	2.9 ± 0.2 ^a^	3.7 ± 0.7 ^b^
22:6n-3 (DHA)	19.2 ± 2.0 ^a^	21.3 ± 1.1 ^ab^	23.0 ± 1.1 ^b^	27.5 ± 1.1 ^c^
EPA + DHA	28.1 ± 2.6 ^a^	30.8 ± 1.0 ^ab^	33.1 ± 1.2 ^bc^	39. 5 ± 1.6 ^c^
Sum n-3 ****	35.4 ± 2.3 ^a^	37.9 ± 1.0 ^ab^	39.9 ± 1.1 ^bc^	45.7 ± 0.9 ^c^
Sum PUFA	54.6 ± 1.7 ^a^	56.0 ± 0.7 ^ab^	56.6 ± 1.0 ^bc^	57.7 ± 0.9 ^c^
n6/n3	0.6 ± 0.1 ^c^	0.5 ± 0.0 ^bc^	0.4 ± 0.0 ^ab^	0.3 ± 0.0 ^a^
TFA	21.6 ±1.6	20.4 ± 2.4	20.8 ± 2.0	21.3 ± 1.0

* Includes 14:0, 15:0, 17:0 and 20:0. ** Includes 16:1n-9, 18:1n-11, 20:1n-11, 20:1n-7, 22:1n-11, 22:1n-9 and 24:1n-9. *** Includes 22:4n-6 and 22:5n-6. **** Includes 18:4n-3, 21:5n-3, 24:5n-3 and 24:6n-3.

**Table 5:** Liver fatty acid composition of neutral lipids in Atlantic salmon fed diets with increasing dietary EPA + DHA (mg/g). Three cages per diet group and 4 fish sampled per cage (*n* = 12). Different letters denote significant statistical differences (*p* < 0.05, Kruskal–Wallis). Numbers are the mean with standard deviation.

	Diet 1.0	Diet 1.3	Diet 1.6	Diet 3.5
16:0	2.3 ± 1.5	2.3 ± 1.9	1.3 ± 0.5	0.9 ± 0.3
18:0	2.1 ± 1.6 ^b^	1.8 ± 1.3 ^b^	0.9 ± 0.5 ^ab^	0.5 ± 0.2 ^a^
Sum SFA *	5.3 ± 3.5 ^b^	4.9 ± 3.8 ^b^	2.7 ± 1.2 ^ab^	1.7 ± 0.7 ^a^
16:1n-7	0.9 ± 0.7	0.9 ± 0.8	0.5 ± 0.3	0.4 ± 0.2
18:1n-9	39.1 ± 32.4 ^b^	34.5 ± 26.6 ^b^	18.4 ± 9.3 ^b^	6.5 ± 3.2 ^a^
18:1n-7	2.5 ± 2.0 ^b^	2.2 ± 1.6 ^b^	1.3 ± 0.6 ^ab^	0.7 ± 0.3 ^a^
20:1n-9	4.0 ± 3.4 ^b^	3.5 ± 2.7 ^b^	2.0 ± 0.9 ^b^	0.9 ± 0.5 ^a^
Sum MUFA **	47.6 ± 39.2 ^b^	42.1 ± 32.4 ^b^	22.7 ± 11.3 ^b^	9.1 ± 4.4 ^a^
18:2n-6	11.0 ± 10.2 ^b^	9.4 ± 7.1 ^b^	5.0 ± 2.6 ^b^	1.8 ± 0.8 ^a^
20:2n-6	1.7 ± 1.5 ^b^	1.6 ± 1.3 ^b^	0.9 ± 0.5 ^b^	0.4 ± 0.2 ^a^
20:3n-6	0.52 ± 0.46 ^c^	0.31 ± 0.22 ^bc^	0.09 ± 0.15 ^b^	0.04 ± 0.02 ^a^
20:4n-6 (ARA)	0.5 ± 0.4 ^ab^	0.6 ± 0.5 ^b^	0.4 ± 0.2 ^ab^	0.2 ± 0.1 ^a^
Sum n-6 ***	13.9 ± 12.6 ^b^	12.0 ± 9.2 ^b^	6.5 ± 3.3 ^b^	2.4 ± 1.1 ^a^
18:3n-3	3.4 ± 2.9 ^b^	3.3 ± 2.6 ^b^	1.9 ± 0.9 ^b^	0.7 ± 0.3 ^a^
20:4n-3	0.4 ± 0.4	0.4 ± 0.4	0.2 ± 0.1	0.2 ± 0.1
20:5n-3 (EPA)	0.5 ± 0.2	0.6 ± 0.4	0.4 ± 0.1	0.6 ± 0.2
22:5n-3	0.1 ± 0.1 ^a^	0.1 ± 0.1 ^a^	0.1 ± 0.0 ^a^	0.3 ± 0.1 ^b^
22:6n-3 (DHA)	0.4 ± 0.1 ^a^	0.5 ± 0.2 ^a^	0.4 ± 0.1 ^ab^	0.6 ± 0.2 ^b^
EPA + DHA	0.9 ± 0.4	1.1 ± 0.6	0.9 ± 0.1	1.2 ± 0.3
Sum n-3 ****	5.0 ± 3.7	5.1 ± 3.8	3.2 ± 1.2	2.5 ± 0.9
Sum PUFA	19.0 ± 16.3 ^b^	17.1 ± 13.0 ^b^	9.7 ± 4.6 ^ab^	4.9 ± 2.0 ^a^
n6/n3	2.5 ± 0.6 ^c^	2.3 ± 0.2 ^bc^	1.9 ± 0.3 ^ab^	1.0 ± 0.2 ^a^
TFA	72.6 ± 59.3 ^b^	65.0 ± 49.7 ^b^	35.6 ± 17.3 ^b^	16.1 ± 7.2 ^a^

* Includes 14:0, 15:0, 17:0 and 20:0. ** Includes 16:1n-9, 18:1n-11, 20:1n-11, 20:1n-7, 22:1n-11, 22:1n-9 and 24:1n-9. *** Includes 18:3n-6 and 22:4n-6. **** Includes 18:4n-3, 21:5n-3 and 24:5n-3.

**Table 6:** Core findings in the metabolomics data from livers of Atlantic salmon fed increasing dietary levels of EPA and DHA.

	Pathway	Metabolites	Core Finding
Energy production	Tricarboxylic acid cycle (TCA)	Decreased citrate, cis- aconitate and isocitrate and increased succinate with lower dietary EPA and DHA.	Indicates less use of FA oxidation as input into TCA cycle, and then a shift to using BCAA, PPP and creatine instead when salmon is fed a lower dietary EPA and DHA.
	Pentose Phosphate Pathway	Increased ribulose-5-phosphate (intermediate in PPP) and galactonate (feeds into the PPP) in fish fed less EPA and DHA.	
	BCAA metabolism	Increased levels of BCAA, dipeptides containing BCAA (protein degradants), and acyl carnitines involved in BCAA metabolism in fish given lower dietary EPA and DHA.	
	Creatine metabolism	Higher levels of these metabolites with lower dietary EPA and DHA	
Transport of FA into mitochondria	Carnitine metabolism	Increased levels of carnitine and deoxycarnitine in fish fed less EPA and DHA.	Suggests some dysfunction with the mitochondrial β-oxidation.
	Acyl carnitines	Higher occurrence with lower dietary EPA and DHA, particularly fish fed diet 1.6.	
Phospholipid metabolism	Phospholipid degradants	Higher content of PL degradants with lower dietary EPA and DHA.	Implies an altered PL metabolism with lowered dietary EPA and DHA. Can possibly disturb the mitochondrial membrane.
	Lysophospholipids	More of almost all lysophospholipids in liver of fish given diets low in EPA and DHA.	

**Table 7:** Metabolites included in the tricarboxylic acid cycle (TCA), pentose phosphate pathway and branched amino acid catabolism in Atlantic salmon fed diets with increasing dietary EPA + DHA. Triplicate cages for diets 1.6 and 3.5 and duplicate cages for diet 1.0 with five fish sampled per cage (*n* = 15 and *n* = 10, respectively). Data are presented as the fold change between pairwise comparisons of diet groups, e.g., for the comparison of diet 1.0/diet 3.5, a number below one would indicate less of the metabolite in diet group 1.0, while a number above one would indicated the opposite. Red indicates significantly higher and green significantly lower (*p* < 0.05), while pink indicate *p*-values between 0.05 and 0.1.

Pathway	Biochemical Name	Fold Change		
		Diet 1.0/Diet 3.5	Diet 1.6/Diet 3.5	Diet 1.0/Diet 1.6
TCA Cycle	citrate	0.77	1.32	0.58
	aconitate [cis or trans]	0.83	1.56	0.53
	isocitrate	0.64	1.09	0.59
	succinate	1.47	1.10	1.33
Fatty acid metabolism	acetyl-CoA	0.93	0.90	1.03
Pentose Phosphate Pathway	ribulose 5-phosphate	1.83	0.97	1.89
Fructose, Mannose and Galactose Metabolism	galactonate	1.53	1.48	1.04
Leucine, Isoleucine and Valine Metabolism	leucine	1.10	1.01	1.09
	isoleucine	1.09	1.02	1.06
	valine	1.18	1.06	1.11
	glycylisoleucine	1.56	1.05	1.48
Dipeptide	glycylleucine	1.78	1.12	1.59
	glycylvaline	2.13	1.23	1.74
	isoleucylglycine	5.03	1.21	4.14
	lysylleucine	2.73	1.23	2.23
	prolylglycine	2.00	1.31	1.53
	valylglutamine	3.75	1.31	2.86
	valylglycine	6.17	1.15	5.36
	valylleucine	3.40	1.20	2.84
	leucylglutamine	4.53	1.21	3.75
Fatty Acid Metabolism (also BCAA Metabolism)	butyrylcarnitine (C4)	1.41	1.69	0.83
	propionylcarnitine (C3)	1.48	1.33	1.12
Creatine Metabolism	guanidinoacetate	2.16	1.07	2.01
	creatine	1.06	1.20	0.89
	creatinine	1.28	1.17	1.09

**Table 8:** Metabolites in carnitine metabolism, ketone bodies and metabolism in the synthesis of CoA in Atlantic salmon fed diets with increasing dietary EPA + DHA. Triplicate cages for diets 1.6 and 3.5 and duplicate cages for diet 1.0 with five fish sampled per cage (*n* = 15 and *n* = 10, respectively). Data are presented as the fold change between pairwise comparisons of diet groups, e.g., for the comparison diet 1.0/diet 3.5, a number below one would indicate less of the metabolite in diet group 1.0, while a number above one would indicate the opposite. Red indicates significantly higher. (*p* < 0.05).

Pathway	Biochemical Name	Fold Change		
		Diet 1.0/Diet 3.5	Diet 1.6/Diet 3.5	Diet 1.0/Diet 1.6
Carnitine Metabolism	deoxycarnitine	1.13	1.37	0.83
	carnitine	1.23	1.32	0.93

**Table 9:** Metabolites in tryptophan metabolism and eicosanoids in Atlantic salmon fed diets with increasing dietary EPA + DHA. Triplicate cages for diets 1.6 and 3.5 and duplicate cages for diet 1.0 with five fish sampled per cage (*n* = 15 and *n* = 10, respectively). Data are presented as the fold change between pairwise comparisons of diet groups, e.g., for the comparison diet 1.0/diet 3.5, a number below one would indicate less of the metabolite in diet group 1.0, while a number above one would indicate the opposite. Red indicates significantly higher and green significantly lower (*p* < 0.05).

Pathway	Biochemical Name	Fold Change		
		Diet 1.0/Diet 3.5	Diet 1.6/Diet 3.5	Diet 1.0/Diet 1.6
Eicosanoid	5-HEPE	0.29	0.33	0.88
Tryptophan metabolism	kynurenine	1.70	2.10	0.81
	kynurenate	1.03	1.90	0.54

**Table 10:** qPCR primer sequences, their accession number and primer efficiency.

Gene	Forward	Reverse	GenBank Accession Number	Efficiency
Reference genes				
*arp*	GAAAATCATCCAATTGCTGGATG	CTTCCCACGCAAGGACAGA	AY255630	84%
*b-actin*	CCAAAGCCAACAGGGAGAA	AGGGACAACACTGCCTGGAT	BG933897	109%
*ef1ab*	TGCCCCTCCAGGATGTCTAC	CACGGCCCACAGGTACTG	AF321836	111%
Target genes				
*aco*	CACTGCCAGGTGTGGTGGTA	GGAATTGTACGTTCTCCAATTTCA	DQ364432	97%
*apob100*	TGCAGAGACCTTTAAGTTCATTCA	TGTGCAGTGGTTGCCTTGAC	gi:854619	124%
*cact*	GTTCGCTGTCTGCTTCTTCG	TACTTCACCTCCCCTTTGGC	BT044930.1	87%
*cd36*	GGATGAACTCCCTGCATGTGA	TGAGGCCAAAGTACTCGTCGA	AY606034	113%
*cpt1*	CTTTGGGAAGGGCCTGATC	CATGGACGCCTCGTACGTTA	AM230810	92%
*cpt2*	TGCTCAGCTAGCGTTCCATATG	AGTGCTGCAGGACTCGTATGTG	BG934647	102%
*ppara*	TCTCCAGCCTGGACCTGAAC	GCCTCGTAGACGCCGTACTT	NM001123560	107%
*plin2*	CCACTCTGCCTCGCAAATCT	GGGTAAAAGGGACCTACCAGC	XM_014206726.1	100%

*arp*—acidic ribosomal protein; *ef1ab*—elongation factor 1ab; *aco*—acyl-CoA oxidase, *apob100*—apolipoprotein B 100; *cact*—carnitine acylcarnitine translocase; *cpt*—carnitine palmitoyltransferase; *ppara*—peroxisome proliferator-activated receptor α; *plin2*—perilipin 2.

## Data Availability

All data presented in this study are available within the paper, Appendix A or the Supplementary Materials.

## Supplementary Materials

The full metabolomics dataset can be downloaded at: https://www.mdpi.com/article/10.3390/metabo12020159/s1.

## Appendix A

**Table A1:** Full analysed FA composition in mg/g in liver polar and neutral lipids of Atlantic salmon fed feeds with increasing dietary EPA + DHA. Triplicate cages per diet group, and 4 fish sampled per cage (*n* = 12). Different letters denote significant statistical differences (*p* < 0.05 one-way ANOVA with Tukey’s HSD post hoc). Numbers are the mean with standard deviation.

	Polar Lipids				Neutral Lipids			
	Diet 1.0	Diet 1.3	Diet 1.6	Diet 3.5	Diet 1.0	Diet 1.3	Diet 1.6	Diet
14:0	0.1 ± 0.0 a	0.1 ± 0.0 a	0.1 ± 0.0 a	0.2 ± 0.0 b	0.5 ± 0.4	0.5 ± 0.4	0.3 ± 0.1	0.2 ± 0.1
16:0	2.4 ± 0.3 a	2.3 ± 0.3 a	2.3 ± 0.3 a	2.7 ± 0.2 b	2.3 ± 1.5	2.3 ± 1.9	1.3 ± 0.5	0.9 ± 0.3
18:0	1.3 ± 0.2	1.2 ± 0.2	1.3 ± 0.1	1.4 ± 0.1	2.1 ± 1.6 b	1.8 ± 1.3 b	0.9 ± 0.5 ab	0.5 ± 0.2 a
20:0	0.05 ± 0.01	0.05 ± 0.01	0.05 ± 0.02	0.05 ± 0.01	0.14 ± 0.14 b	0.11 ± 0.07 b	0.06 ± 0.03 b	0.02 ± 0.00 a
Sum SFA	3.9 ± 0.5	3.7 ± 0.4	3.8 ± 0.4	4.3 ± 0.3	5.3 ± 3.5 b	4.9 ± 3.8 b	2.7 ± 1.2 ab	1.7 ± 0.7 a
16:1n-9	0.07 ± 0.01 b	0.06 ± 0.01 b	0.06 ± 0.00 b	0.06 ± 0.00 a	0.3 ± 0.2 b	0.3 ± 0.2 b	0.2 ± 0.1 b	0.1 ± 0.0 a
16:1n-7	0.1 ± 0.0 a	0.1 ± 0.0 a	0.1 ± 0.0 a	0.2 ± 0.0 b	0.9 ± 0.7	0.9 ± 0.8	0.5 ± 0.3	0.4 ± 0.2
18:1n-11	0.02 ± 0.00 a	0.01 ± 0.00 a	0.02 ± 0.00 a	0.07 ± 0.02 b	0.03 ± 0.02 b	0.03 ± 0.01 b	0.03 ± 0.01 b	0.1 ± 0.01 a
18:1n-9	4.5 ± 0.5 b	4.0 ± 0.6 b	3.7 ± 0.4 b	2.7 ± 0.3 a	39.1 ± 32.4 b	34.5 ± 26.6 b	18.4 ± 9.3 b	6.5 ± 3.2 a
18:1n-7	0.4 ± 0.0	0.4 ± 0.1	0.4 ± 0.0	0.5 ± 0.0	2.5 ± 2.0 b	2.2 ± 1.6 b	1.3 ± 0.6 ab	0.7 ± 0.3 a
20:1n-11	<0.1	<0.1	<0.1	<0.1	0.05 ± 0.04	0.04 ± 0.03	0.04 ± 0.02	0.06 ± 0.04
20:1n-9	0.5 ± 0.1 ab	0.4 ± 0.1 a	0.5 ± 0.1 ab	0.6 ± 0.1 b	4.0 ± 3.4 b	3.5 ± 2.7 b	2.0 ± 0.9 b	0.9 ± 0.5 a
20:1n-7	0.01 ± 0.00 a	0.01 ± 0.00 a	0.02 ± 0.01 a	0.03 ± 0.00 b	0.10 ± 0.09 b	0.09 ± 0.07 b	0.05 ± 0.03 ab	0.03 ± 0.02 a
22:1n-11	0.01 ± 0.00 a	0.01 ± 0.00 a	0.01 ± 0.00 a	0.02 ± 0.00 b	0.1 ± 0.1	0.1 ± 0.1	0.1 ± 0.0	0.1 ± 0.1
22:1n-9	<0.1	<0.1	<0.1	<0.1	0.2 ± 0.2 b	0.2 ± 0.1 b	0.1 ± 0.1 ab	0.1 ± 0.0 a
24:1n-9	0.02 ± 0.01	0.01 ± 0.01	0.01 ± 0.00	0.01 ± 0.01	0.1 ± 0.1 b	0.1 ± 0.1 b	0.1 ± 0.0 b	0.1 ± 0.0 a
Sum MUFA	5.7 ± 0.5 b	5.0 ± 0.7 ab	4.8 ± 0.5 ab	4.1 ± 0.4 a	47.6 ± 39.2 b	42.1 ± 32.4 b	22.7 ± 11.3 b	9.1 ± 4.4 a
18:2n-6	2.3 ± 0.3 b	2.2 ± 0.4 b	1.9 ± 0.3 b	1.1 ± 0.1 a	11.0 ± 10.2 b	9.4 ± 7.1 b	5.0 ± 2.6 b	1.8 ± 0.8 a
18:3n-6	<0.1	<0.1	<0.1	<0.1	0.10 ± 0.07 c	0.05 ± 0.03 bc	0.02 ± 0.01 ab	0.01 ± 0.0 a
20:2n-6	0.5 ± 0.1 ab	0.5 ± 0.1 ab	0.5 ± 0.1 b	0.4 ± 0.1 a	1.7 ± 1.5 b	1.6 ± 1.3 b	0.9 ± 0.5 b	0.4 ± 0.2 a
20:3n-6	0.5 ± 0.1 c	0.4 ± 0.1 b	0.3 ± 0.1 b	0.1 ± 0.0 a	0.52 ± 0.46 c	0.31 ± 0.22 bc	0.09 ± 0.15 b	0.04 ± 0.02 a
20:4n-6 (ARA)	0.7 ± 0.1 a	0.6 ± 0.1 a	0.7 ± 0.1 a	0.8 ± 0.1 b	0.5 ± 0.4 ab	0.6 ± 0.5 b	0.4 ± 0.2 ab	0.2 ± 0.1 a
22:5n-6	0.08 ± 0.01	0.09 ± 0.01	0.09 ± 0.01	0.09 ± 0.00	<0.1	<0.1	<0.1	<0.1
Sum n-6	4.1 ± 0.3 b	3.7 ± 0.5 b	3.4 ± 0.4 b	2.5 ± 0.1 a	13.9 ± 12.6 b	12.0 ± 9.2 b	6.5 ± 3.3 b	1.1 ± 2.4 a
18:3n-3	0.6 ± 0.1 b	0.5 ± 0.1 b	0.5 ± 0.1 b	0.3 ± 0.1 a	3.4 ± 2.9 b	3.3 ± 2.6 b	1.9 ± 0.9 b	0.7 ± 0.3 a
18:4n-3	0.03 ± 0.0 b	0.02 ± 0.0 ab	0.02 ± 0.02 b	0.01 ± 0.00 b	0.16 ± 0.11 b	0.11 ± 0.07 b	0.06 ± 0.03 ab	0.03 ± 0.01 a
20:4n-3	0.3 ± 0.0 b	0.2 ± 0.0 b	0.2 ± 0.0 ab	0.2 ± 0.0 a	0.4 ± 0.4	0.4 ± 0.4	0.2 ± 0.1	0.2 ± 0.1
20:5n-3 (EPA)	1.9 ± 0.3 a	2.0 ± 0.2 a	2.1 ± 0.2 a	2.6 ± 0.2 b	0.5 ± 0.2	0.6 ± 0.4	0.4 ± 0.1	0.6 ± 0.2
21:5n-3	0.02 ± 0.00 a	0.02 ± 0.00 a	0.02 ± 0.00 a	0.03 ± 0.00 b	0.02 ± 0.01	0.02 ± 0.02	0.02 ± 0.01	0.03 ± 0.01
22:5n-3	0.6 ± 0.1 a	0.6 ± 0.1 a	0.6 ± 0.1 a	0.8 ± 0.2 b	0.1 ± 0.1 a	0.1 ± 0.1 a	0.1 ± 0.0 a	0.3 ± 0.1 b
22:6n-3 (DHA)	4.1 ± 0.6 a	4.4 ± 0.4 a	4.8 ± 0.4 a	5.9 ± 0.3 b	0.4 ± 0.1 a	0.5 ± 0.2 a	0.4 ± 0.1 ab	0.6 ± 0.2 b
24:5n-3	0.02 ± 0.00 a	0.02 ± 0.00 a	0.02 ± 0.01 a	0.04 ± 0.04 b	0.03 ± 0.02 a	0.04 ± 0.06 ab	0.03 ± 0.02 a	0.06 ± 0.02 b
24:6n-3	0.03 ± 0.00	0.04 ± 0.01	0.03 ± 0.01	0.02 ± 0.01	<0.1	<0.1	<0.1	<0.1
EPA + DHA	6.1 ± 0.8 a	6.3 ± 0.6 a	6.9 ± 0.6 a	8.4 ± 0.5 b	0.9 ± 0.4	1.1 ± 0.6	0.9 ± 0.1	1.2 ± 0.3
Sum n-3	7.7 ± 0.9 a	7.7 ± 0.8 a	8.2 ± 0.8 ab	9.7 ± 0.4 b	5.0 ± 3.7	5.1 ± 3.8	3.2 ± 1.2	2.5 ± 0.9
Sum PUFA	11.8 ± 1.1	11.5 ± 1.3	11.7 ± 1.2	12.3 ± 0.5	19.0 ± 16.3 b	17.1 ± 13.0 b	9.7 ± 4.6 ab	4.9 ± 2.0 a
n6/n3	0.6 ± 0.1 c	0.5 ± 0.0 bc	0.4 ± 0.0 ab	0.3 ± 0.0 a	2.5 ± 0.6 c	2.3 ± 0.2 bc	1.9 ± 0.3 ab	1.0 ± 0.2 a
n3/n6	1.8 ± 0.2 a	2.1 ± 0.1 ab	2.4 ± 0.2 b	3.8 ± 0.2 c	0.5 ± 0.2 a	0.4 ± 0.0 a	0.6 ± 0.2 ab	1.1 ± 0.3 b
Sum fat	21.6 ± 1.6	20.5 ± 2.4	20.8 ± 2.0	21.3 ± 1.0	72.6 ± 59.3 b	65.0 ± 49.7 b	35.6 ± 17.3 b	16.1 ± 7.2 a

**Table A2:** Fully analysed FA composition in % of TFA in liver polar and neutral lipids of Atlantic salmon fed feeds with increasing dietary EPA + DHA. Triplicate cages per diet group and 4 fish sampled per cage (*n* = 12). Different letters denote significant statistical differences (*p* < 0.05 one-way ANOVA with Tukey’s HSD post hoc). Numbers are the mean with standard deviation.

	Polar Lipids				Neutral Lipids			
	Diet 1.0	Diet 1.3	Diet 1.6	Diet 3.5	Diet 1.0	Diet 1.3	Diet 1.6	Diet 3.5
14:0	0.4 ± 0.1	0.5 ± 0.0	0.6 ± 0.1	0.9 ± 0.1	0.8 ± 0.1 a	0.8 ± 0.1 a	0.9 ± 0.1 a	1.5 ± 0.2 b
16:0	10.9 ± 0.9 a	11.0 ± 0.6 a	11.2 ± 0.7 a	12.5 ± 1.0 b	3.8 ± 1.2 a	3.6 ± 0.4 a	3.8 ± 0.7 a	6.1 ± 1.1 b
18:0	6.2 ± 0.7	6.1 ± 0.4	6.1 ± 0.4	6.3 ± 0.6	3.0 ± 0.6	2.8 ± 0.4	2.6 ± 0.6	2.9 ± 0.6
20:0	0.2 ± 0.0	0.2 ± 0.0	0.3 ± 0.1	0.3 ± 0.1	0.2 ± 0.0 b	0.2 ± 0.0 b	0.2 ± 0.0 b	0.1 ± 0.0 a
Sum SFA	18.1 ± 1.2 a	18.1 ± 0.7 a	18.5 ± 0.8 a	20.5 ± 1.1 b	8.0 ± 1.5 a	7.6 ± 0.7 a	7.7 ± 1.1 a	11.1 ± 1.7 b
16:1n-9	0.4 ± 0.1	0.3 ± 0.1	0.3 ± 0.1	0.2 ± 0.0	1.3 ± 0.3 a	1.3 ± 0.2 a	1.4 ± 0.1 a	2.6 ± 0.2 b
16:1n-7	0.5 ± 0.1 a	0.5 ± 0.1 a	0.5 ± 0.0 a	0.8 ± 0.1 b	1.3 ± 0.3 a	1.3 ± 0.2 a	1.4 ± 0.1 a	2.6 ± 0.2 b
18:1n-11	0.1 ± 0.0 a	0.1 ± 0.0 a	0.1 ± 0.0 a	0.4 ± 0.1 b	<0.1	<0.1	<0.1	0.1 ± 0.1
18:1n-9	20.9 ± 2.7 c	19.4 ± 0.9 bc	17.9 ± 1.0 ab	12.4 ± 1.3 a	52.9 ± 3.1 c	52.9 ± 0.6 bc	50.9 ± 2.6 b	39.4 ± 2.8 a
18:1n-7	2.0 ± 0.2 a	2.0 ± 0.1 a	2.0 ± 0.1 a	2.3 ± 0.1 b	3.4 ± 0.2 a	3.5 ± 0.2 a	3.6 ± 0.1 a	4.2 ± 0.2 b
20:1n-11	<LOQ	<LOQ	<LOQ	<LOQ	<0.1	<0.1	<0.1	<0.1
20:1n-9	2.2 ± 0.3 a	2.1 ± 0.3 a	2.3 ± 0.4 ab	2.6 ± 0.6 b	5.3 ± 0.7	5.4 ± 0.4	5.5 ± 0.4	5.4 ± 0.8
20:1n-7	<0.1	<0.1	<0.1	<0.1	0.1 ± 0.0 a	0.1 ± 0.0 ab	0.2 ± 0.1 ab	0.2 ± 0.0 b
22:1n-11	<0.1	<0.1	<0.1	<0.1	0.2 ± 0.1 a	0.2 ± 0.0 a	0.2 ± 0.1 a	0.8 ± 0.3 b
22:1n-9	<0.1	<0.1	<0.1	<0.1	0.3 ± 0.0	0.3 ± 0.1	0.3 ± 0.1	0.3 ± 0.1
24:1n-9	<0.1	<0.1	<0.1	<0.1	0.2 ± 0.1	0.2 ± 0.1	0.3 ± 0.1	0.3 ± 0.1
Sum MUFA	26.3 ± 2.9 c	24.6 ± 1.1 bc	23.3 ± 1.0 ab	19.3 ± 1.6 a	64.4 ± 3.6 bc	64.6 ± 0.5 b	63.1 ± 2.8 ab	55.1 ± 3.9 a
18:2n-6	10.9 ± 1.1 c	10.5 ± 0.7 bc	9.2 ± 0.6 b	5.3 ± 0.5 a	14.4 ± 1.2 b	14.4 ± 0.6 b	13.9 ± 0.8 b	10.9 ± 0.9 a
18:3n-6	<0.1	<0.1	<0.1	<0.1	<0.1	<0.1	<0.1	<0.1
20:2n-6	2.1 ± 0.3 ab	2.3 ± 0.3 b	2.3 ± 0.3 b	1.8 ± 0.3 a	2.3 ± 0.3	2.4 ± 0.3	2.5 ± 0.2	2.3 ± 0.2
20:3n-6	2.5 ± 0.4 d	1.7 ± 0.3 c	1.2 ± 0.2 b	0.4 ± 0.1 a	0.7 ± 0.2 c	0.5 ± 0.1 b	0.4 ± 0.1 b	0.3 ± 0.1 a
20:4n-6 (ARA)	3.1 ± 0.4 a	3.1 ± 0.2 a	3.3 ± 0.2 ab	3.9 ± 0.3 b	0.8 ± 0.1 a	0.9 ± 0.1 ab	1.1 ± 0.2 b	1.5 ± 0.2 c
22:5n-6	0.3 ± 0.1 a	0.4 ± 0.0 ab	0.4 ± 0.0 b	0.4 ± 0.0 b	<0.1	<0.1	<0.1	<0.1
Sum n-6	19.2 ± 0.9 d	18.1 ± 0.7 c	16.6 ± 0.8 b	12.0 ± 0.4 a	18.4 ± 1.4 b	18.3 ± 0.8 b	18.0 ± 0.8 b	15.1 ± 0.9 a
18:3n-3	2.6 ± 0.5 b	2.6 ±0.3 b	2.5 ± 0.3 b	1.4 ± 0.4 a	4.6 ± 0.4 ab	5.0 ± 0.4 bc	5.2 ± 0.3 c	4.2 ± 0.6 a
18:4n-3	At LOQ	At LOQ	At LOQ	At LOQ	0.3 ± 0.1	0.2 ± 0.0	0.2 ± 0.0	0.2 ± 0.1
20:4n-3	1.3 ± 0.2 c	1.2 ± 0.2 ab	1.1 ± 0.1 b	0.8 ± 0.1 a	0.7 ± 0.3 a	0.6 ± 0.1 a	0.7 ± 0.1 a	1.2 ± 0.1 b
20:5n-3 (EPA)	8.9 ± 0.8 a	9.5 ± 0.6 ab	10.1 ± 0.6 b	12.0 ± 0.7 c	1.0 ± 0.8 a	1.0 ± 0.3 a	1.5 ± 0.9 ab	3.8 ± 0.8 b
21:5n-3	At LOQ	At LOQ	At LOQ	At LOQ	<0.1	<0.1	<0.1	<0.1
22:5n-3	2.8 ± 0.3 a	2.8 ± 0.2 a	2.9 ± 0.2 a	3.7 ± 0.7 b	0.2 ± 0.3 a	0.2 ± 0.0 a	0.3 ± 0.2 a	1.7 ± 0.4 b
22:6n-3 (DHA)	19.2 ± 2.0 a	21.3 ± 1.1 ab	23.0 ± 1.1 b	27.5 ± 1.1 c	1.1 ± 1.5 a	1.0 ± 0.4 a	1.6 ± 1.2 a	4.6 ± 1.8 b
24:5n-3	0.1 ± 0.0 a	0.1 ± 0.0 ab	0.1 ± 0.0 ab	0.2 ± 0.0 b	0.1 ± 0.0 a	0.1 ± 0.0 a	0.1 ± 0.0 a	0.4 ± 0.1 b
24:6n-3	0.2 ± 0.0 b	0.2 ± 0.0 b	0.1 ± 0.1 ab	0.1 ± 0.0 a	<0.1	<0.1	<0.1	<0.1
EPA + DHA	28.1 ± 2.6 a	30.8 ± 1.0 ab	33.1 ± 1.2 bc	39. 5 ± 1.6 c	2.1 ± 2.3 a	2.0 ± 0.7 a	3.1 ± 2.1 a	8.4 ± 2.5 b
Sum n-3	35.4 ± 2.3 a	37.9 ± 1.0 ab	39.9 ± 1.1 bc	45.7 ± 0.9 c	7.9 ± 2.9 a	8.1 ± 0.6 ab	9.7 ± 2.4 b	16.4 ± 2.9 bc
Sum PUFA	54.6 ± 1.7 a	56.0 ± 0.7 ab	56.6 ± 1.0 bc	57.7 ± 0.9 c	26.3 ± 2.8 a	26.5 ± 2.4 a	27.7 ± 2.4 ab	31.5 ± 2.5 b
n6/n3	0.6 ± 0.1 c	0.5 ± 0.0 bc	0.4 ± 0.0 ab	0.3 ± 0.0 a	2.5 ± 0.6 c	2.3 ± 0.2 bc	1.9 ± 0.3 ab	1.0 ± 0.2 a
n3/n6	1.8 ± 0.2 a	2.1 ± 0.1 ab	2.4 ± 0.2 b	3.8 ± 0.2 c	0.5 ± 0.2 a	0.4 ± 0.0 a	0.6 ± 0.2 ab	1.1 ± 0.3 b *
Sum fat	21.6 ±1.6	20.4 ± 2.4	20.8 ± 2.0	21.3 ± 1.0	72.6 ± 59.3 b	65.0 ± 49.7 b	35.6 ± 17.3 b	16.1 ± 7.1 a

**Table A3:** Various acyl carnitines in the livers of Atlantic salmon fed diets with increasing contents of EPA + DHA. Triplicate cages for diets 1.6 and 3.5, duplicate cages for diet 1.0 with five fish sampled per cage (*n* = 15 and *n* = 10, respectively). Data are presented as fold change between pairwise comparisons of diet groups, e.g., for the comparison diet 1.0/diet 3.5, a number below one would indicate less of the metabolite in diet group 1.0, while a number above one would indicate the opposite. Red indicates significantly higher and green significantly lower (*p* < 0.05), while pink and light green indicate *p*-values between 0.05 and 0.1.

Pathway	Biochemical Name	Fold Change		
		Diet 1.0/Diet 3.5	Diet 1.6/Diet 3.5	Diet 1.0/Diet 1.6
Fatty Acid Metabolism (Acyl Carnitine, Short Chain)	acetylcarnitine (C2)	1.25	1.43	0.87
	isocaproylcarnitine	0.53	0.83	0.64
Fatty Acid Metabolism (Acyl Carnitine, Medium Chain)	hexanoylcarnitine (C6)	1.75	1.87	0.94
	cis-3,4-methyleneheptanoylcarnitine	0.39	0.96	0.40
	decanoylcarnitine (C10)	1.65	1.52	1.09
	laurylcarnitine (C12)	1.73	1.52	1.14
Fatty Acid Metabolism (Acyl Carnitine, Long Chain Saturated)	palmitoylcarnitine (C16)	0.88	1.42	0.62
	margaroylcarnitine (C17)	0.64	1.17	0.55
	stearoylcarnitine (C18)	1.21	1.69	0.72
	arachidoylcarnitine (C20)	1.25	1.46	0.86
	behenoylcarnitine (C22)	1.47	1.39	1.06
	lignoceroylcarnitine (C24)	1.11	1.22	0.91
Acid Metabolism (Acyl Carnitine, Monounsaturated)	palmitoleoylcarnitine (C16:1)	0.62	0.98	0.63
	oleoylcarnitine (C18:1)	1.14	2.03	0.56
	eicosenoylcarnitine (C20:1)	1.18	1.89	0.62
	erucoylcarnitine (C22:1)	0.62	0.71	0.87
	ximenoylcarnitine (C26:1)	0.64	0.79	0.81
Fatty Acid Metabolism (Acyl Carnitine, Polyunsaturated)	linoleoylcarnitine (C18:2)	1.12	1.75	0.64
	linolenoylcarnitine (C18:3)	0.97	1.46	0.67
	dihomo-linoleoylcarnitine (C20:2)	1.19	1.99	0.60
	arachidonoylcarnitine (C20:4)	0.87	1.11	0.78
	dihomo-linolenoylcarnitine (C20:3n3 or 6)	2.48	2.56	0.97
	docosadienoylcarnitine (C22:2)	1.12	1.61	0.70
	docosapentaenoylcarnitine (C22:5n3)	0.64	1.01	0.63
	docosahexaenoylcarnitine (C22:6)	0.94	1.21	0.77
Fatty Acid Metabolism (Acyl Carnitine, Hydroxy)	(S)-3-hydroxybutyrylcarnitine	1.16	1.37	0.84
	3-hydroxyhexanoylcarnitine (1)	1.36	1.29	1.05
	3-hydroxyoctanoylcarnitine (1)	1.75	1.58	1.11
	3-hydroxyoctanoylcarnitine (2)	1.54	1.47	1.05
	3-hydroxydecanoylcarnitine	2.36	1.75	1.35
	3-hydroxypalmitoylcarnitine	1.54	1.47	1.05
	3-hydroxyoleoylcarnitine	1.58	1.88	0.84

**Table A4:** Metabolites in phospholipid metabolism and lysophospholipids in Atlantic salmon fed diets with increasing dietary EPA + DHA. Triplicate cages for diets 1.6 and 3.5 and duplicate cages for diet 1.0 with five fish sampled per cage (*n* = 15 and *n* = 10, respectively). Data are presented as the fold change between pairwise comparisons of diet groups, e.g., for the comparison diet 1.0/diet 3.5, a number below one would indicate less of the metabolite in diet group 1.0, while a number above one would indicate the opposite. Red indicates significantly higher and green significantly lower (*p* < 0.05), while pink and light green indicate *p*-values between 0.05 and 0.1.

Pathway	Biochemical Name	Fold Change		
		Diet 1.0/Diet 3.5	Diet 1.6/Diet 3.5	Diet 1.0/Diet 1.6
Phospholipid metabolism	glycerophosphoethanolamine	1.46	1.39	1.05
	glycerophosphoserine	1.25	1.35	0.93
	glycerophosphoinositol	1.34	1.18	1.14
Lysophospholipid	1-palmitoyl-GPC (16:0)	1.06	0.96	1.10
	2-palmitoyl-GPC (16:0)	1.62	1.03	1.57
	1-palmitoleoyl-GPC (16:1)	0.71	0.72	0.99
	2-palmitoleoyl-GPC (16:1)	0.65	0.73	0.88
	1-stearoyl-GPC (18:0)	1.41	1.31	1.08
	1-oleoyl-GPC (18:1)	1.42	1.38	1.03
	1-linoleoyl-GPC (18:2)	2.09	1.80	1.16
	1-linolenoyl-GPC (18:3)	2.01	1.91	1.05
	1-dihomo-linolenoyl-GPC (20:3n3 or 6)	3.30	1.98	1.67
	2-dihomo-linolenoyl-GPC (20:3n3 or 6)	4.62	2.85	1.62
	1-omega-arachidonoyl-GPC (20:4n3)	1.42	1.21	1.17
	1-arachidonoyl-GPC (20:4n6)	0.81	0.72	1.13
	1-eicosapentaenoyl-GPC (20:5)	0.70	0.76	0.93
	1-lignoceroyl-GPC (24:0)	1.81	1.77	1.02
	1-palmitoyl-GPE (16:0)	0.88	0.87	1.01
	1-stearoyl-GPE (18:0)	1.18	1.17	1.01
	2-stearoyl-GPE (18:0)	1.28	0.98	1.31
	1-oleoyl-GPE (18:1)	1.19	1.17	1.02
	1-linoleoyl-GPE (18:2)	1.43	1.26	1.13
	2-dihomo-linolenoyl-GPE (20:3n3 or 6)	4.20	2.34	1.79
	1-dihomo-linolenoyl-GPE (20:3n3 or 6)	2.36	1.67	1.41
	1-omega-arachidonoyl-GPE (20:4n3)	1.24	1.06	1.17
	1-arachidonoyl-GPE (20:4n6)	1.07	0.86	1.25
	1-eicosapentaenoyl-GPE (20:5)	1.27	1.10	1.16
	1-palmitoyl-GPS (16:0)	0.96	1.04	0.92
	1-stearoyl-GPS (18:0)	1.63	2.06	0.79
	1-oleoyl-GPS (18:1)	1.20	1.38	0.86
	1-palmitoyl-GPG (16:0)	1.08	0.65	1.66
	1-palmitoyl-GPI (16:0)	1.27	0.90	1.41
	1-stearoyl-GPI (18:0)	1.42	1.05	1.35
	1-oleoyl-GPI (18:1)	2.01	1.26	1.59
	1-linoleoyl-GPI (18:2)	2.56	1.60	1.60
	1-arachidonoyl-GPI (20:4)	1.00	0.81	1.24
